# Diarrhea as a Potential Cause and Consequence of Reduced Gut Microbial Diversity Among Undernourished Children in Peru

**DOI:** 10.1093/cid/ciz905

**Published:** 2019-09-17

**Authors:** Saba Rouhani, Nicholas W Griffin, Pablo Peñataro Yori, Jeanette L Gehrig, Maribel Paredes Olortegui, Mery Siguas Salas, Dixner Rengifo Trigoso, Lawrence H Moulton, Eric R Houpt, Michael J Barratt, Margaret N Kosek, Jeffrey I Gordon

**Affiliations:** 1 Johns Hopkins Bloomberg School of Public Health, Baltimore, Maryland; 2 Edison Family Center for Genome Sciences and Systems Biology, Washington University School of Medicine, Washington; 3 Center for Gut Microbiome and Nutrition Research, St. Louis, Missouri; 4 Asociación Benéfica Preferred Reporting Items for Systematic Reviews and Meta-analyses, Iquitos, Peru; 5 University of Virginia, Charlottesville, Virginia

**Keywords:** diarrhea, stunting, microbiota

## Abstract

**Background:**

Detrimental effects of diarrhea on child growth and survival are well documented, but details of the underlying mechanisms remain poorly understood. Recent evidence demonstrates that perturbations to normal development of the gut microbiota in early life may contribute to growth faltering and susceptibility to related childhood diseases. We assessed associations between diarrhea, gut microbiota configuration, and childhood growth in the Peruvian Amazon.

**Methods:**

Growth, diarrhea incidence, illness, pathogen infection, and antibiotic exposure were assessed monthly in a birth cohort of 271 children aged 0–24 months. Gut bacterial diversity and abundances of specific bacterial taxa were quantified by sequencing 16S rRNA genes in fecal samples collected at 6, 12, 18, and 24 months. Linear and generalized linear models were used to determine whether diarrhea was associated with altered microbiota and, in turn, if features of the microbiota were associated with the subsequent risk of diarrhea.

**Results:**

Diarrheal frequency, duration, and severity were negatively associated with bacterial diversity and richness (*P* < .05). Children born stunted (length-for-age *z*-score [LAZ] ≤ −2) who were also severely stunted (LAZ ≤ −3) at the time of sampling exhibited the greatest degree of diarrhea-associated reductions in bacterial diversity and the slowest recovery of bacterial diversity after episodes of diarrhea. Increased bacterial diversity was predictive of reduced subsequent diarrhea from age 6 to 18 months.

**Conclusions:**

Persistent, severe growth faltering may reduce the gut microbiota's resistance and resilience to diarrhea, leading to greater losses of diversity and longer recovery times. This phenotype, in turn, denotes an increased risk of future diarrheal disease and growth faltering.

## Teaser

In this study, we report longitudinal associations between diarrhea and significant, lasting changes to the gut microbiota among members of a cohort of Peruvian children aged 0–24 months and describe how these effects interact with linear growth faltering (stunting) and subsequent risk of enteric disease.

**(See the Editorial Commentary by Colin Stine on pages 1008–9 and the Major Article by Rouhani et al on pages 1000–7.)**

The cycle between diarrhea and undernutrition is well described but remains poorly understood. Children with high burdens of enteric infection experience compromised growth in early life, resulting in impaired immunity and vulnerability to future diarrhea. This cycle constitutes the leading cause of long-term disability among children aged <5 years; its impact on intestinal function, linear growth, cognition, and school performance [1–4] underlie a loss of human potential in 200 million children worldwide [5]. The persistence of this cycle despite numerous water, sanitation, hygiene, and nutrition interventions [6] highlights a need for a better understanding of its underlying mechanisms.

A growing body of evidence illustrates the role of the gut microbiota in development of immune and metabolic functions and other facets of postnatal growth [7]. Children with acute malnutrition possess gut microbial profiles distinct from those that exhibit healthy growth, and growth-faltering phenotypes can be transmitted by transplanting microbiota from undernourished children into germ-free mice and ameliorated by exposure to components of a healthy donor microbiota [8–10]. These observations provide preclinical evidence that altered microbiota composition is a cause, not simply an effect, of undernutrition and that establishment of the microbiota in early childhood is linked to healthy growth. Many studies have also documented changes in the gut microbiota before, during, and after diarrheal episodes [11–13]. Moreover, case-control comparisons from the Global Enterics Multicenter Study (GEMS) suggest that moderate-to-severe diarrhea leads to reduced bacterial diversity and altered microbiota composition in children [14]. These observations suggest that children who suffer multiple episodes of diarrhea in early life may suffer impairments in gut microbiota development that could contribute to repeated diarrhea or persistent illness and growth faltering and that pre- and probiotic interventions may improve their outcomes [15]. However, much of the research informing this hypothesis has utilized animal models or cross-sectional studies of humans with small sample sizes, limiting a more thorough analysis of the short- and long-term consequences of disrupted gut microbial community development in early life. In this study, we examine the associations between diarrhea, bacterial diversity and richness, and stunting in a longitudinal birth cohort of 271 children aged 0–24 months in the Peruvian Amazon in order to better understand the potential role of the microbiota in the cycle of diarrhea and undernutrition in low- and middle-income countries.

## METHODS

### Study Setting and Design

This study was nested in the Etiology, Risk Factors, and Interactions of Enteric Infection and Malnutrition and the Consequences for Child Health and Development (MAL-ED) Study. MAL-ED birth cohort studies were conducted at 8 sites with high burdens of childhood undernutrition and diarrhea [16]. MAL-ED Peru is located in the lowlands of the Amazon basin in Santa Clara de Nanay, where access to water and sanitation is lower than elsewhere in the country and where diarrheal disease prevalence and mortality among those aged <5 years are nearly 3 times the national average [17].

**Figure 1.**
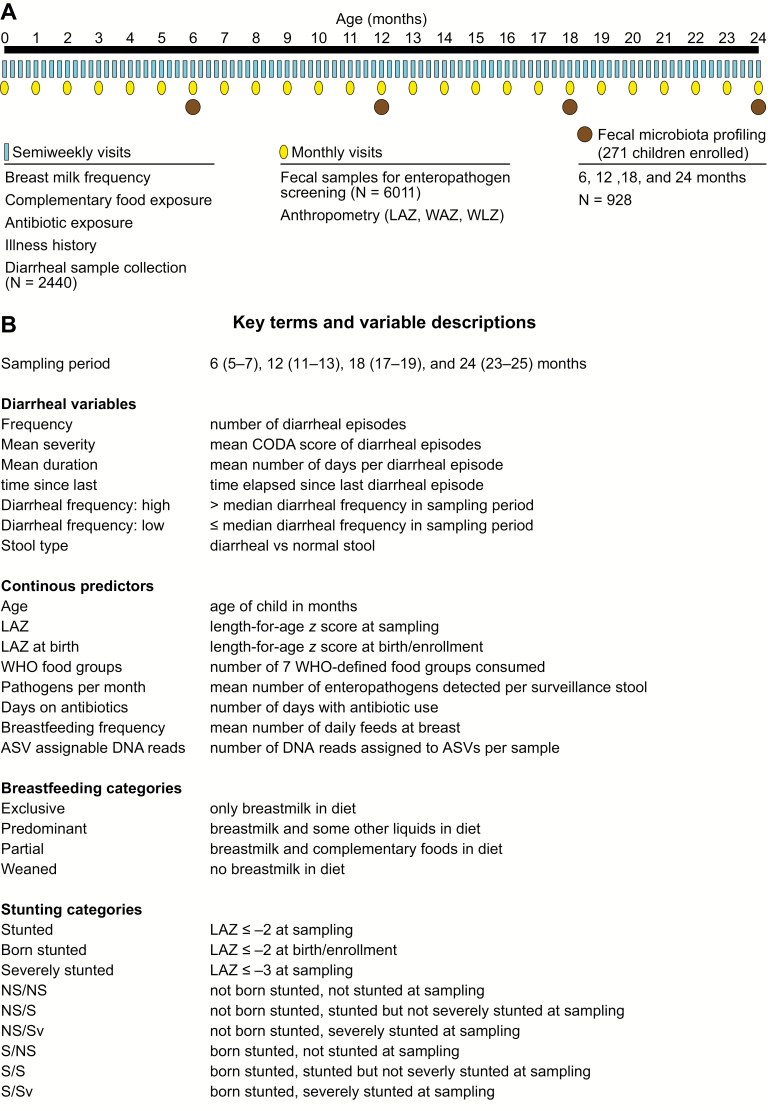
Study design. *A*, Diagram of the timeline of data collection at semiweekly visits, as well and sample collection. *B*, Key terms and variables are described. Abbreviations: ASV, amplicon sequence variant; CODA, community diarrheal assessment tool; NS/NS, not born stunted, not stunted at the time of sampling; LAZ, length-for-age *z*-score; S/Sv, stunted and severely stunted at sampling; WAZ, weight-for-age *z*-score; WHO, World Health Organization; WLZ, weight-for-length *z*-score.

Figure 1A summarizes the study design. Children were enrolled between November 2009 and February 2012 (n = 271; 146 males, 125 females). Study protocols are reported elsewhere [18, 19]. Briefly, birth date, sex, information about initiation of breastfeeding, and anthropometric data were collected at enrollment within 17 days of birth. Children contributed monthly fecal surveillance samples for analysis of asymptomatic infection, and active surveillance for diarrhea was conducted twice weekly during which additional diarrheal specimens were collected for diagnostic analysis. Data on fever, antibiotic exposure, and breastfeeding were ascertained during these visits to generate a continuous history of diet and illness. Anthropometrics were measured monthly. A total of 6011 surveillance fecal samples, representing a mean of 22 samples per child (92.4% completeness), and 2440 diarrheal samples were analyzed. Routine and diarrheal fecal specimens were screened using previously published bacterial culture methods, immunoassays for protozoa and viruses, and microscopy to assay for the presence of >40 enteropathogens [20].

Routine surveillance specimens from children who remained in the study at postnatal months 6 (n = 271 children), 12 (n = 250), 18 (2 = 231), and 24 (n = 213) were used for analysis of fecal microbiota. A total of 934 (96.8%) stool samples were available for sequencing. Variable region 4 of bacterial 16S rRNA genes in fecal DNA samples was amplified using published primers [8] and polymerase chain reaction conditions. Amplicons were sequenced using an Illumina MiSeq instrument. The resulting 250-nucleotide reads were oriented, trimmed of primer sequences, and paired using “bbduk.sh” and “repair.sh” tools in the bbtools (37.02) software package (https://sourceforge.net/projects/bbmap/).

Using R (3.5.1) [21], we removed chimeric sequences, identified taxa as amplicon sequence variants (ASV, or individual DNA sequences) and constructed a phylogenetic tree (packages DADA2[1.8.0] [22]; ClustalOmega' msa[1.14.0] [23]; phangorn[2.4.0] [24]]. Metrics of community diversity and richness were estimated using the phyloseq [25] and picante(1.7) [26] packages. ASVs were assigned taxonomy using the RDP Naive Bayesian Classifier algorithm and the GreenGenes (13.8) [27] training set; assignment is displayed in Supplementary Table 1. Eleven samples were omitted (6 due to low number of reads [<1000] during sequencing, 3 outliers with >4.5 standard deviations (SDs) greater than the next highest diversity and richness estimates in their age bins, and 2 samples that were the only specimens collected during exclusive breastfeeding); the final number of samples included in analyses of microbiota was 923.

### Analytic Approach

Key terms are defined in Figure 1B. Diarrhea was defined as passage of ≥3 loose stools in a 24-hour period, and frequency was defined as the cumulative number of distinct episodes (separated by 2 diarrhea-free days) experienced prior to each time point. Severity was assessed using a community diarrheal assessment score based on presence and duration of fever, vomiting, anorexia, liquid stools, and maximum stool output [28]. Breastfeeding frequency was characterized by the mean number of cumulative feeds per 24 hours and categorized as exclusive, predominant, partial, or weaned. Dietary diversity was calculated as cumulative exposure to 7 World Health Organization (WHO)–defined food groups recommended for complementary feeding [29]. Standardized length-for-age *z*-scores (LAZs) were calculated using WHO guidelines [21]. Bacterial diversity was assessed using Shannon's diversity index (ShanD), which is influenced by both the number and distribution of different species, and Simpson's diversity index (SimpD), which describes the sum of the squared proportional abundances of all ASVs within a given sample. Bacterial richness was measured using the Chao1 index (Chao1), a capture-recapture–based estimate of the number of ASVs in a sample, and phylogenetic diversity (PD), the total branch length of the phylogeny represented by ASVs per sample for assignment.

### Statistical Analyses

Linear regression models with generalized estimating equations were used to adjust for within-child correlations and identify factors associated with prior diarrheal incidence. Associations between diarrhea and bacterial community metrics were investigated using linear mixed-effects models, with stepwise model simplification using the Akaike information criterion (AIC). Models initially included age, LAZ at enrollment and time of sampling, history of antibiotic use, breastfeeding, complementary feeding, and infection. Unequal sequencing depth was controlled for by including the number of V4-16S rDNA reads assigned to ASVs per sample. Two models were considered: frequency-only, with diarrheal episodes as the sole diarrhea-related predictor, and full model, with episodes, mean severity, mean duration, and days since last diarrhea as predictors among children who only experience diarrhea. Variables and interactions considered prior to stepwise simplification are available in Supplementary Table 2A. Continuous predictors were centered and scaled by their SDs. Stunting categories were defined by being stunted (LAZ ≤ −2) at birth and not stunted (LAZ > −2), stunted (LAZ ≤ −2), or severely stunted (LAZ ≤ −3) at the time of sampling. ShanD and PD were scaled and centered, Chao1 was log-transformed, and SimpD was logit-transformed. To explore whether observed associations endured over time, we refit simplified models in a subcohort of children without recent exposure (no diarrhea or antibiotics for ≥1 month at time of sampling; frequency-only: N = 69 children, 161 samples; full: N = 66 children, 133 samples).

A 2-stage approach was adopted to identify ASVs associated with diarrhea and stunting in each sampling period: Poisson models with quasi-generalized linear model corrections were used to identify associations with relative abundances and logistic regressions were used to identify associations with the presence/absence of particular ASVs. In each period, Poisson models were performed for ASVs present in at least half of the samples with a mean relative abundance of 0.1%, and logistic regressions were run for ASVs present in 10%–90% of the samples. Presence and abundance were compared between children with low vs high diarrheal frequency (≤median vs >median for each age bin). Overall *P* values for each model were calculated by comparison to an intercept-only model and were corrected for multiple tests using the Benjamini-Hochberg method.

Finally, analyses to predict future diarrheal frequency were performed using Poisson and negative binomial generalized linear models, followed by simplification by AIC. ASV-assignable DNA reads per sample was intentionally retained in all models. Chao1 was the only measure of bacterial diversity used in order to reduce the collinearity of predictor variables. All continuous predictors were scaled and centered, including the counts of particular ASVs.

## RESULTS

### Description of the Population

A total of 260 children (95.9%) experienced diarrhea (Table 1); mean and median time to the first episode were 5.3 and 4.6 months, respectively. Diarrheal incidence increased with age during the first year of life and decreased thereafter; by 24 months, children had experienced a mean of 8.8 episodes lasting, on average, 4 days each. Seventy-seven percent of diarrheal samples (n = 1887) were pathogen-positive, with up to 7 enteropathogens detected in a sample (Table 1). Forty-four percent of asymptomatic surveillance stools were pathogen-positive, with a mean of 0.71 (range, 0–6) pathogens detected per sample.

**Table 1.** Lifetime History of Diarrhea, Growth, Breastfeeding, and Gut Bacterial Diversity Indices at Each Quarter of Life in the Etiology, Risk Factors, and Interactions of Enteric Infection and Malnutrition and the Consequences for Child Health and Development Peruvian Birth Cohort

	Age, months			
	6	12	18	24
Representation of breastfeeding categories, % (n)				
Exclusively breastfed	0	0	0	0
Predominantly breastfed	3 (8)	0	0	0
Partially breastfed	96 (254)	94 (228)	52 (114)	16 (32)
Weaned	1 (2)	5 (13)	48 (107)	84 (173)
Linear growth (LAZ) and incidence of stunting				
Mean LAZ (95% CI)	−1.3 (−1.4 to −1.2)	−1.6 (−1.7 to −1.5)	−1.8 (−2.0 to −1.7)	−1.9 (−2.0 to −1.8)
% (n) stunted; 95% CI	22.2 (60); 17.2 to 27.2	31.5 (78); 25.6 to 37.3	42.9 (96); 36.3 to 49.4	40.1 (87); 34.2 to 47.5
% (n) severely stunted; 95% CI	4.1 (11); 1.7 to 6.4	5.3 (13); 2.5 to 8.0	10.3 (23); 6.3 to 14.3	10.8 (23); 6.6 to 15.0
Diarrheal disease, mean (95% CI)				
No. distinct diarrheal episodes	2.0 (1.7 to 2.2)	4.5 (4.1 to 4.9)	7.1 (6.5 to 7.7)	8.8 (8.0 to 9.5)
Days per diarrheal episode	4.7 (4.2 to 5.1)	4.2 (4.0 to 4.5)	3.9 (3.7 to 4.1)	3.7 (3.5 to 3.9)
Diarrheal severity score (community diarrheal assessment)	1.8 (1.7 to 2.0)	2.2 (2.0 to 2.4)	2.3 (2.2 to 2.5)	2.2 (2.1 to 2.3)
% diarrheal stools with ≥1 pathogen detected	56.5 (51.3 to 61.7)	71.7 (68.2 to 75.4)	78.0 (75.1 to 80.8)	80.5 (78.0 to 83.0)
No. pathogens per diarrheal sample	0.8 (.7 to .9)	1.3 (1.2 to 1.1)	1.5 (1.4 to 1.6)	1.6 (1.5 to 1.7)
Bacterial diversity indices, mean (95% CI)				
Shannon's diversity	1.03 (.97 to 1.09)	1.94 (1.86 to 2.02)	2.73 (2.64 to 2.82)	3.29 (3.21 to 3.37)
Simpson's diversity	0.43 (.40 to .45)	0.69 (.67 to .71)	0.84 (.82 to .85)	0.91 (.89 to .92)
Phylogenetic diversity	7.1 (6.8 to 7.4)	12.6 (12.0 to 13.2)	18.2 (17.4 to 19.0)	23.4 (22.5 to 24.3)
Chao1 index	22.1 (21.0 to 23.2)	46.8 (43.5 to 50.0)	82.0 (76.9 to 87.2)	116.6 (110.4 to 122.9)

n = 271.

Abbreviations: CI, confidence interval; LAZ, length-for-age *z*-score.

All infants received breast milk, and limited variation in breastfeeding habits was observed (Table 1). The mean duration of exclusive breastfeeding and time to weaning were 41 days and 19.5 months, respectively. By age 2 years, 69% of children had been exposed to all WHO complementary food groups. A total of 262 (97%) children were exposed to antibiotics from age 0 to 24 months, with a mean cumulative duration of exposure of 53 days. Overall, 181 (66.8%) children were stunted, and 22.1% were severely stunted on at least 1 monthly visit. Thirty-four children (12.6%) were stunted at enrollment (Table 1).

Exclusive breastfeeding was protective against diarrhea in the first year of life (predominant odds ratio [OR:], 2.5; *P* ≤ .001; partial OR, 2.4; *P* < .001; weaned OR, 3.3; *P* = .011), as was continued exposure to breastmilk in the second year (partial vs weaned: OR, 1.2; *P* = .045). From 0 to 24 months, each diarrheal episode was associated with a reduction in LAZ of 0.01 (*β* = −0.01; *P* = .002), after adjustment for age, breastfeeding, and LAZ at enrollment.

### Factors Influencing the Gut Microbiota

**Figure 2.**
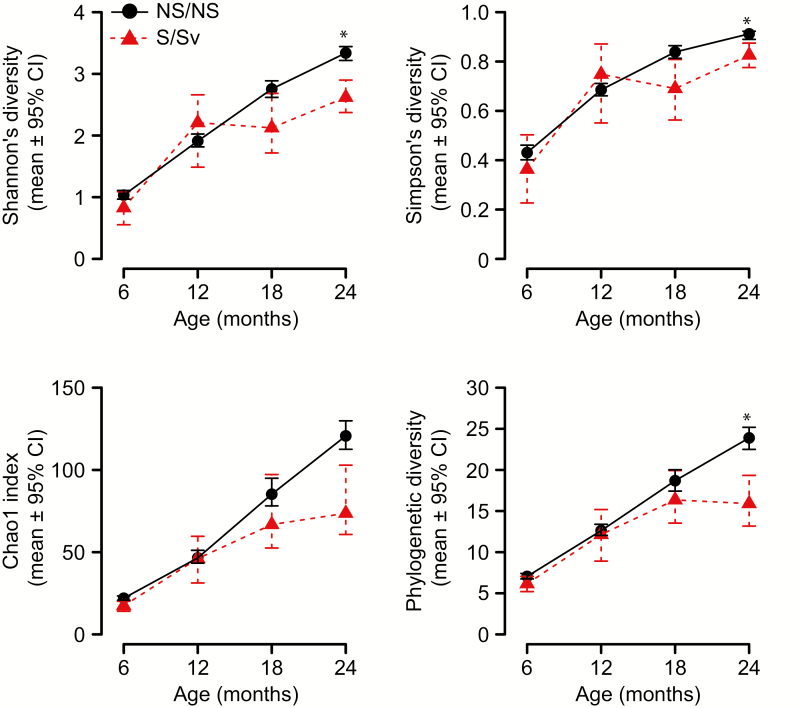
Bacterial diversity and richness across age by stunting category. Line graphs showing mean values for (*A*) Shannon's diversity, (*B*) Simpson's diversity, (*C*) the Chao1 index, and (*D*) phylogenetic diversity in each sampling period (6, 12, 18, and 24 months) for children born NS/NS vs children born S/Sv. Abbreviations: CI, confidence interval; NS/NS, not born stunted, not stunted at the time of sampling; S/Sv, stunted and severely stunted at sampling.

**Figure 3.**
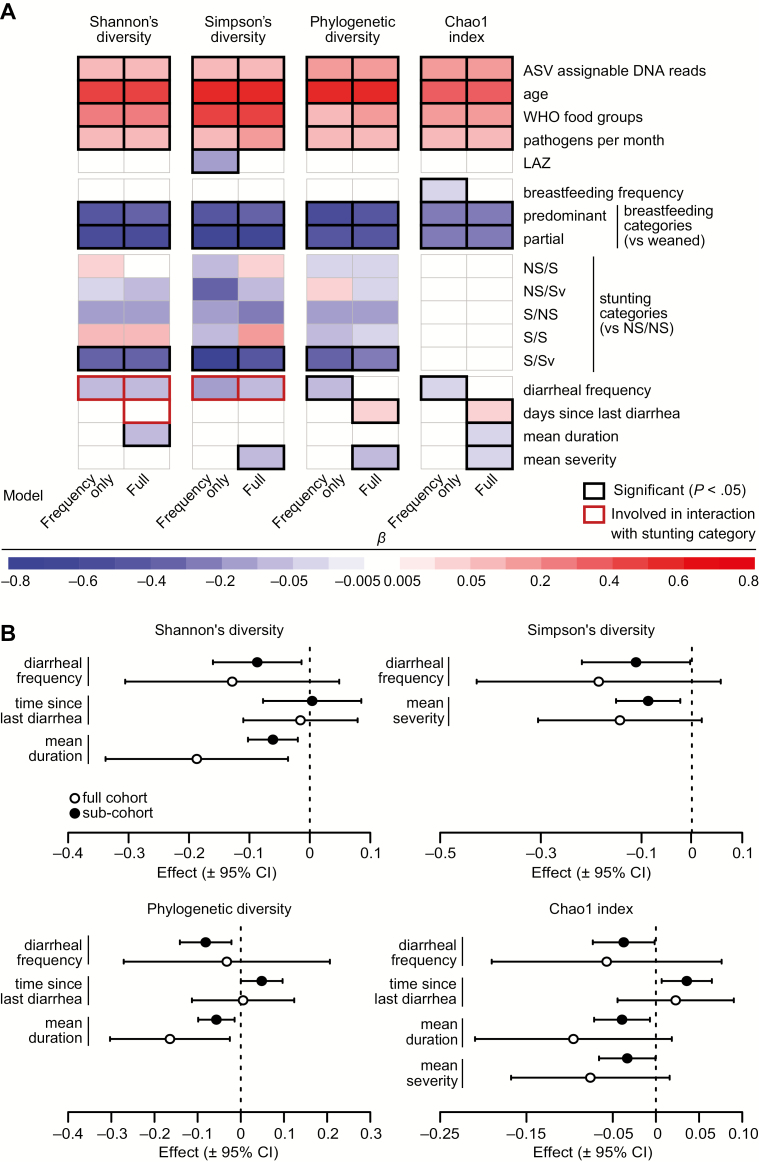
Results of linear mixed-effects models testing associations between metrics of gut bacterial diversity and richness and measures of growth, feeding history, and health. *A*, Heat map showing the effects of diet, the indicated health parameters, and diarrheal variables on fecal bacteria diversity metrics, as estimated in the frequency-only and full models. The coefficients represent changes in standard deviations for Shannon's diversity and phylogenetic diversity, log values for the Chao1 index, and logit-transformed values for Simpson's diversity expected from a 1-standard deviation increase in continuous predictors and differences between the indicated group for breastfeeding and stunting categories. For effects of diarrheal variables involved in interactions with stunting category (in red boxes), the coefficient shown represents the overall mean effect. For the effects in all stunting categories, see Figure 3 and Supplementary Figure 1. *B*, Forest plots showing the effects of diarrheal frequency, duration, severity, and time since diarrhea on gut bacterial diversity metrics in the full cohort and subcohort analyses. Effects are shown in the same scale as in the heat map of panel A. Abbreviations: ASV, amplicon sequence variant; CI, confidence interval; LAZ, length-for-age *z*-score; NS/NS, not born stunted, not stunted at the time of sampling; NS/S, not born stunted, stunted but not severely stunted at sampling; NS/Sv, not born stunted, severely stunted at sampling; S/NS, born stunted/not stunted at sampling; S/S, born stunted, stunted but not severely stunted at sampling; S/Sv, stunted and severely stunted at sampling; WHO, World Health Organization.

Bacterial diversity and richness increased significantly with age, dietary diversity, and asymptomatic carriage of enteropathogens and decreased with exposure to breast milk (Figures 2, 3A, Table 2, Supplementary Table 2A–2I). Children born stunted who became severely stunted during follow-up exhibited compromised trajectories of diversity and richness acquisition compared with children who were neither born stunted nor were stunted at the time of sampling (Figure 2).

**Table 2.** Effects of Diarrheal Exposures and Covariates on Measures of Bacterial Diversity and Richness in the Fecal Microbiota of the Etiology, Risk Factors, and Interactions of Enteric Infection and Malnutrition and the Consequences for Child Health and Development Peruvian Birth Cohort

	Mean Change in Standardized (βz) and Actual (β) Gut Microbial Diversity Measures (βz [95% Confidence Interval]; β)			
Predictor Variable	Shannon's Diversity	Simpson's Diversity^a^	Phylogenetic Diversity	Chao1 Index^b^
Total V4-16S rDNA reads assigned to amplicon sequence variants per fecal sample (per 1000 reads)	0.006 (.002 to .01); 0.006**	0.006 (0 to .011)*	0.013 (.009 to .017); 0.101***	0.013 (.01 to .016); 1.3***
Age (months)	0.072 (.06 to .085); 0.076***	0.082 (.063 to .1)***	0.089 (.074 to .103); 0.704***	0.056 (.047 to .064); 5.7***
World Health Organization food groups	0.089 (.062 to .117); 0.094***	0.169 (.126 to .212)***	0.036 (.009 to .063); 0.288**	0.073 (.054 to .092); 7.6***
Pathogens per month	0.223 (.109 to .337); 0.234***	0.282 (.102 to .463)**	0.264 (.148 to .38); 2.087***	0.234 (.147 to .321); 26.4***
LAZ at sampling	^c^	−0.116 (−.223 to −.008)*	^c^	^c^
Breastfeeding frequency	^c^	^c^	^c^	−0.007 (−.014 to 0); −0.7*
Breastfeeding categories (vs weaned)				
Partial	−0.519 (−.631 to −.407); −0.544***	−0.637 (−.803 to −.472)***	−0.47 (−.593 to −.347); −3.721***	−0.272 (−.351 to −.192); −23.8***
Predominant	−0.442 (−.642 to −.241); −0.463***	−0.439 (−.776 to −.102)*	−0.517 (−.716 to −.318); −4.092***	−0.25 (−.395 to −.105); −22.1***
Stunting categories (vs NS/NS)				
NS/S	0.024 (−.069 to .117); 0.025	−0.088 (−.278 to .102)	−0.017 (−.115 to .081); −0.137	^c^
NS/Sv	−0.021 (−.229 to .187); −0.022	−0.337 (−.733 to .058)	0.014 (−.197 to .225); 0.113	^c^
S/NS	−0.118 (−.309 to .074); −0.123	−0.154 (−.461 to .154)	−0.163 (−.38 to .055); −1.287	^c^
S/S	0.067 (−.117 to .252); 0.071	−0.054 (−.363 to .255)	−0.062 (−.252 to .128); −0.49	^c^
S/Sv	−0.319 (−.53 to −.108); −0.334**	−0.783 (−1.197 to −.369)***	−0.307 (−.544 to −.071); −2.433*	^c^
Diarrheal variables (presented as effects across the entire cohort, regardless of involvement in interactions)				
Frequency (no. episodes)	−0.02 (−.03 to −.003); −0.02^d^	−0.02 (−.05 to −.001)^d^	−0.018 (−.03 to −.005); −0.14**	−0.008 (−.016 to 0); −0.8*
Time since last diarrhea (months)^e^	0.001 (−.027 to .029); 0.001^d^	^c^	0.017 (0 to .033); 0.133*	0.012 (.002 to .022); 1.2*
Mean duration (days)^e^	−0.028 (−.046 to −.009); −0.029**	^c^	^c^	−0.018 (−.032 to −.003); −1.8*
Mean severity (community diarrheal assessment)^e^	^c^	−0.07 (−.121 to −.019)**	−0.046 (−.079 to −.012); −0.364**	−0.027 (−.053 to −.001); −2.6*
Effects of diarrheal frequency by stunting category (vs NS/NS)^f^				
NS/S	−0.016 (−.035 to .004); −0.016	−0.022 (−.051 to .007)	^c^	^c^
NS/Sv	0.026 (−.01 to .062); 0.027	0.042 (−.012 to .096)	^c^	^c^
S/NS	−0.031 (−.065 to .002); −0.033	−0.049 (−.1 to .002)	^c^	^c^
S/S	0.016 (−.03 to .062); 0.017	0.028 (−.043 to .099)	^c^	^c^
S/Sv	−0.057 (−.109 to −.006); −0.06*	−0.099 (−.177 to −.021)*	^c^	^c^

Abbreviations: LAZ, length-for-age z-score; NS/NS, not born stunted and not stunted at the time of sampling; NS/S, not born stunted, stunted but not severely stunted at sampling; NS/Sv, not born stunted, severely stunted at sampling; S/NS, born stunted/not stunted at sampling; S/S, born stunted, stunted but not severely stunted at sampling; S/Sv, stunted and severely stunted at sampling.

^a^Estimates for Simpson's diversity are presented in units of logit-transformed Simpson's diversity per unit increase of the predictor variable.

^b^Back-transformed coefficients for Chao1 are interpreted as percent change.

^c^Predictor variable eliminated during Akaike information criterion–based stepwise model selection.

^d^Estimates for effects involved in interactions are presented as mean effects without indicators of significance.

^e^Estimates for time since diarrhea, mean diarrheal duration, and mean diarrheal severity are taken from full models. All others are from frequency-only models.

^f^Stunting categories are written as stunting status at birth/stunting status at sampling: NS = LAZ ≥ −2; S = LAZ < −2); Sv = LAZ < −3.

*, *P* < .05; **, *P* < .01; ***, *P* < .001.

**Figure 4.**
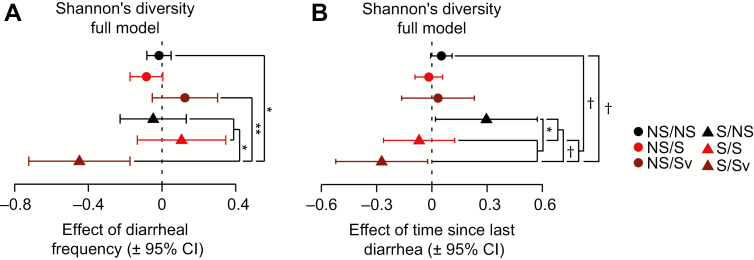
Effects of diarrheal variables on Shannon's diversity by stunting category in full models. *A*, Forest plots showing the effects of diarrheal frequency and (*B*) time since diarrhea on Shannon's diversity, and indicated significant comparisons between groups in post hoc planned linear contrasts. *P* values were corrected using Holm's method. Abbreviations: CI, confidence interval; NS/S, not born stunted, stunted but not severely stunted at sampling; NS/Sv, not born stunted, severely stunted at sampling; S/NS, born stunted/not stunted at sampling; S/S, born stunted, stunted but not severely stunted at sampling; S/Sv, stunted and severely stunted at sampling. ^†^*P* < .1; * *P* < .05; ** *P* < .01.

Associations between predictors and bacterial diversity and richness are shown in Figures 3 and 4 and Supplementary Table 2A–2O. Diarrheal frequency was negatively associated with bacterial diversity and richness in most models; each additional episode of diarrhea was associated with mean decreases of 0.1 and 0.8 in PD and Chao1, respectively (Table 2, Supplementary Table 2H and 2I). Mean diarrheal duration and severity were associated with reduced bacterial diversity and richness and elapsed each month after diarrhea was associated with mean increases of 0.7 in PD and 5.7% in Chao1 (Figure 3A, 3B, Table 2, Supplementary Table 2F–2I). Effects of diarrhea on diversity and richness were similar in the subcohort who experienced ≥1 month without diarrhea (Figure 3B).

Fecal microbiota from S/Sv children (born stunted, severely stunted at sampling) exhibited significantly greater reductions in bacterial diversity (ShanD/SimpD) per diarrheal episode than children not stunted at birth (NS/NS, NS/Sv) or severely stunted at sampling (S/NS and S/S; Figure 4A, Supplementary Figure 1, Table 2, Supplementary Table 2A–2N). S/Sv children had the slowest mean recovery, though this was marginally significant after corrections for multiple comparisons (Figure 4B, Table 2, Supplementary Table 2D and 2E).

**Figure 5.**
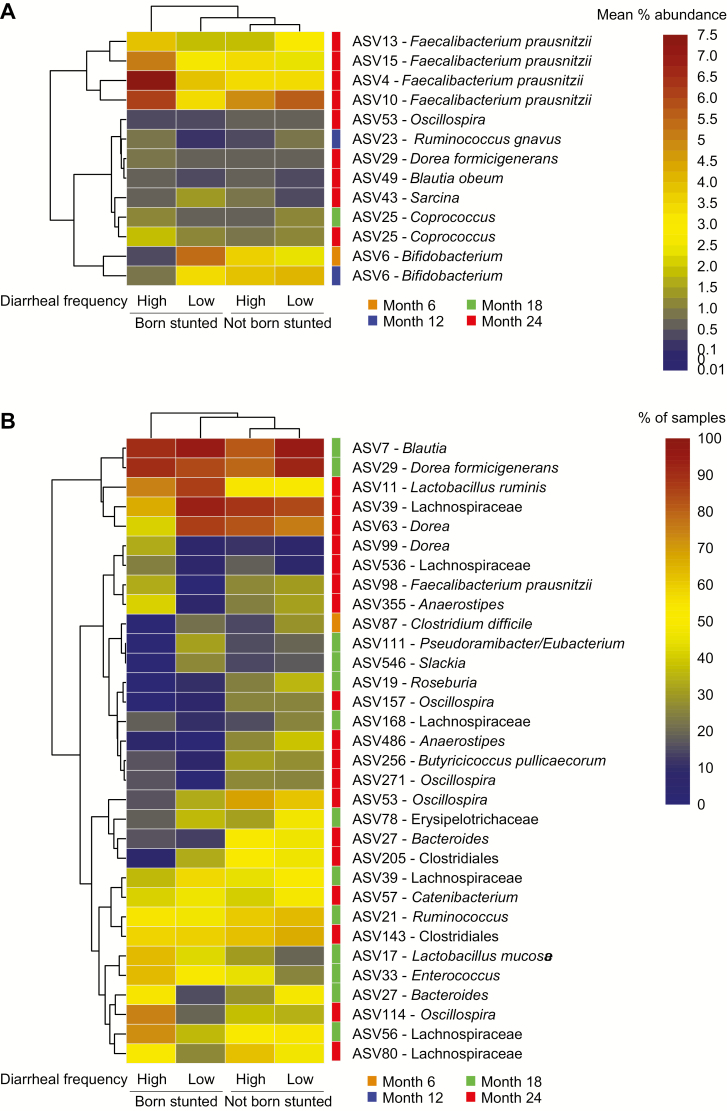
Associations between amplicon sequence variants (ASVs), diarrheal frequency, and stunting at birth. *A*, Heat map showing the mean percent abundances of ASVs in the microbiota of children at the indicated sampling time points (6, 12, 18, and 24 months) by stunting category at birth and diarrheal incidence (low = diarrheal episodes less than or equal to the median frequency for the cohort; high = more than the median frequency of diarrheal episodes). Only ASVs with a mean percent relative abundance of ≥0.5% in at least 1 category are shown. *B*, Data from panel A expressed as mean frequencies of occurrence of ASVs. ASVs with a mean frequency of ≥25% in a group are shown. All ASVs depicted had significant Poisson models or logistic regressions after Benjamini-Hochberg corrections and significant likelihood ratio tests or *F* tests for stunting, diarrheal frequency, or their interaction.

Diarrhea and stunting were also significantly associated with the presence and relative abundances of several bacterial taxa (ASVs; Supplementary Table 3A–3G). Although statistical power to detect interactions was low, analyses revealed that lower diversity at age 24 months in children born stunted was influenced by increased relative abundance of ASVs assigned to *Faecalibacterium prausnitzii* (Figure 5A, Supplementary Table 3D). For example, ASV4 was significantly more prevalent in children born stunted, and several *F. prausnitzii* ASVs (ASV4, ASV10, ASV13, ASV15) had their greatest mean relative abundances in children born stunted and suffering high diarrheal frequency (interaction only significant for ASV13). Probabilities of occurrence of many ASVs at age 24 months were negatively influenced by stunting or high diarrheal frequency (Figure 5B, Supplementary Table 3G), including members of *Bacteroides* (ASV27), *Oscillospira* (ASV53, ASV157, ASV271, ASV1301), Clostridiales (ASV143, ASV205, ASV455), *Ruminococcaceae* (ASV158, ASV804), *Dorea* (ASV281), and *Prevotella* (ASV204). At age 24 months, many ASVs were more likely to be absent in children born stunted than in those not born stunted. These results mirror the reductions in bacterial diversity observed in S/Sv children.

### Incidence of Subsequent Diarrhea

Chao1 was significantly associated with reductions in future diarrhea. A 1-SD increase in Chao1 was associated with mean reductions in diarrheal episodes of 13.6% (*P* = .011) and 12.5% (*P* = .018) from age 6 to 12 months and age 12 to 18 months, respectively (Supplementary Table 4A and 4B). Several ASVs were retained at age 12–18 and 18–24 months, including *Ruminococcus gnavus* (ASV23) and a member of the *Coriobacteriaceae* (ASV18), which both had negative associations with future diarrhea from age 12 to 18 months (Supplementary Table 4B and 4C). From age 18–24 months, *Bifidobacterium* (ASV1) and *Prevotella copri* (ASV9) were positively associated with future diarrhea, and *Blautia* (ASV7) was negatively correlated with future diarrhea.

## DISCUSSION

We describe associations between gut bacterial community composition and diarrhea in a large birth cohort from a tropical, low-income community with a high burden of undernutrition and enteric infections. Patterns identified are consistent with the wider body of literature that demonstrates protective effects of breastfeeding on the risk of diarrheal disease [31] and inverse associations between diarrhea and child growth [32, 33]. We demonstrate that diarrheal frequency, duration, and severity are significantly associated with enduring reductions in fecal bacterial diversity and richness in a community-based sample of children from birth to age 2 years. These data complement recent analyses within the GEMS study and elsewhere [14, 34–36]. Here, we extend these findings using a longitudinal design to demonstrate that perturbations persist beyond symptomatic illness and are apparent in asymptomatic specimens contributed by children with higher lifetime incidence rates of diarrhea. In turn, we observed that reduced bacterial richness was associated with increased subsequent frequency of all-cause diarrhea, after adjustment for breastfeeding frequency and category, asymptomatic pathogen carriage, dietary diversity, antibiotic exposure, and anthropometric status. These data illustrate the classic cycle of diarrhea and undernutrition and present evidence that disruptions to the gut bacterial community may be implicated in driving this cycle experienced by vulnerable children during a critical period for growth and development.

A compelling finding of our study was the detrimental impact of stunting on the development of the microbiota in early life, including its capacity to recover diversity after diarrheal insult. Children who were born stunted, particularly those with severe stunting thereafter, had distinctly compromised trajectories of postnatal acquisition of bacterial diversity and richness and experienced more severe diarrhea-associated reductions in these metrics. Low birth length was also associated with slower recovery of the bacterial community after diarrhea. These findings indicate that persistence and severity of stunting may impact the ability of the gut bacterial community to resist perturbations during insult and recover from them afterward. Taken together, these results illustrate a potential feedback loop between pre- and postnatal linear growth faltering, impaired bacterial community development, and diarrheal disease.

Stunting often begins in utero, and prenatal growth faltering is linked with long-term sequelae typically considered from the perspective of host developmental biology [37, 38]. However, our findings highlight the importance of systematically examining “host” features associated with being born stunted that could disrupt gut microbial community assembly. For example, new methods allow simultaneous, quantitative measurement of numerous plasma protein biomarkers and mediators of physiologic, metabolic, and immunologic processes [39]. Mass spectrometry or nuclear magnetic resonance–based analyses of urine collected from these children provides a way to define their metabolic phenotypes. Applying these tools to infants and their mothers could provide a better understanding of the many dimensions of maternal effects on the infant microbiota, including how maternal nutrition influences the configuration of their infants' microbial communities and the importance of breast milk components, particularly milk oligosaccharides, in determining infant gut microbial community development [40–42].

Results should be viewed in light of study limitations. The cohort design is subject to bias due to differential loss to follow-up. In this riverine community, families often travel regionally due to seasonal factors related to fishing, farming, and flooding; as such, missed observations are a plausible source of bias in this work. Furthermore, we did not have data on a number of maternal factors (eg, diet, antibiotic use) and household factors (eg, human and animal crowding, antimicrobial usage) that have the potential to influence the gut bacterial community. Nonetheless, we note the inclusion of many factors that are not often available for consideration and believe the granular, longitudinal data used provide useful insights.

The potentially causal link between microbiota development and healthy growth necessitates a view of biological “maturation” that considers codevelopment of the gut microbial community and host. Compounded insults of repeated diarrhea and stunting in infants may disrupt normal gut microbiota development, potentially leading to further diarrhea and growth deficits. Given the high prevalence of diarrhea and stunting in these settings and the inadequate impacts of water, sanitation, and hygiene interventions, the value of directed interventions that support establishment of diverse, age-appropriate microbial communities may help promote healthy development during early childhood in resource-poor settings.

## Supplementary Data

Supplementary materials are available at *Clinical Infectious Diseases* online. Consisting of data provided by the authors to benefit the reader, the posted materials are not copyedited and are the sole responsibility of the authors, so questions or comments should be addressed to the corresponding author.

ciz905_suppl_Supplementary_Figure_S1

ciz905_suppl_Supplementary_Legends

ciz905_suppl_Supplementary_Tables

## Notes

***Acknowledgments.*** The authors are grateful to the families of the birth cohort members described in this article for their participation and assistance and the communities of Santa Clara, Santo Tomas, and La Union. The authors thank Marty Meier, Su Deng, Justin Serugo, Kazi Ahsan, J. Hoisington-López, Monica McGrath, and Dennis Lang for technical assistance and the Health Department of Loreto, Peru (DISA-Loreto). V4-16S rDNA sequencing datasets reported in this study are available through the European Nucleotide Archive (study accession number PRJEB28159).

***Disclaimer.*** Study funders had no role in study design, data collection, data analysis, interpretation, or manuscript preparation. The corresponding and senior authors had access to all data used in the analyses described and take final responsibility for publication decisions.

***Financial support.*** Funding was provided by the Bill & Melinda Gates Foundation; the Foundation for the National Institutes of Health; the Fogarty International Center; the Fisher Center for Environmental Infectious Diseases, Johns Hopkins University; and the Ken and Sherrilyn Fisher Foundation (to M. K.).

***Potential conflicts of interest.*** J. G. is a cofounder of Matatu, Inc, a company that characterizes the role of diet-by-microbiota interactions in animal health. All other authors report no potential conflicts. All authors have submitted the ICMJE Form for Disclosure of Potential Conflicts of Interest. Conflicts that the editors consider relevant to the content of the manuscript have been disclosed.
