# Supplementary figures and images for: Diarrhea as a Potential Cause and Consequence of Reduced Gut Microbial Diversity Among Undernourished Children in Peru
Source: Clin Infect Dis. 2019 Sep 17;71(4):989–99. doi: 10.1093/cid/ciz905 (PMC7053391; doi:10.1093/cid/ciz905)

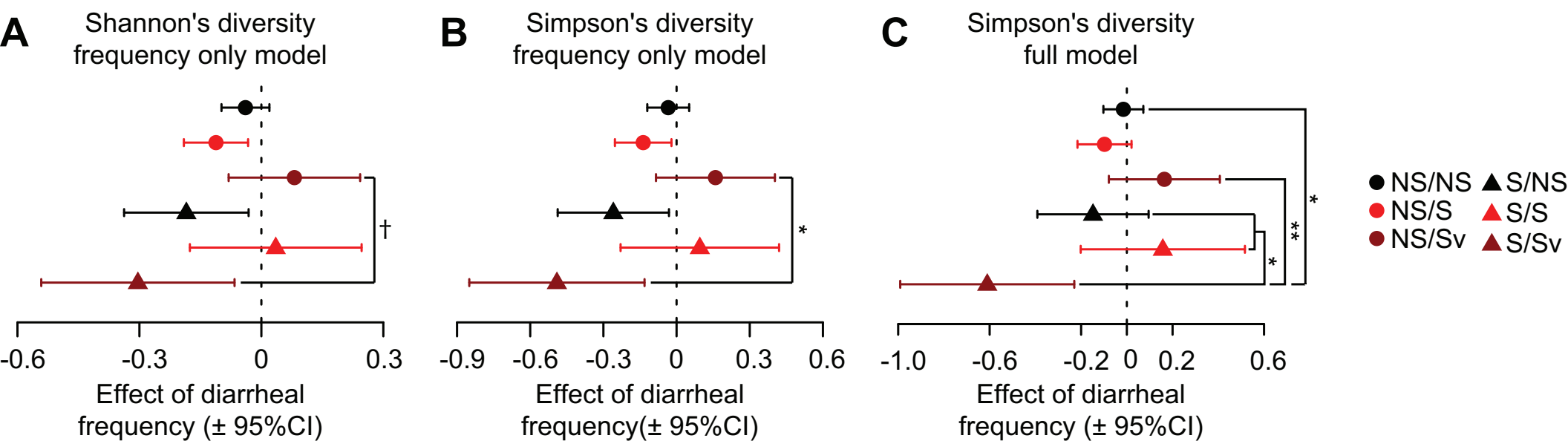

Supplement: ciz905_suppl_Supplementary_Figure_S1 [file ciz905_suppl_supplementary_figure_s1.pdf]
