# Supplementary material for: Diarrhea as a Potential Cause and Consequence of Reduced Gut Microbial Diversity Among Undernourished Children in Peru
Source: Clin Infect Dis. 2019 Sep 17;71(4):989–99. doi: 10.1093/cid/ciz905 (PMC7053391; doi:10.1093/cid/ciz905)
Supplement: ciz905_suppl_Supplementary_Legends [file ciz905_suppl_supplementary_legends.docx]

**Supplementary Figures**

**Figure S1: Effects of diarrheal frequency on Shannon’s and Simpson’s diversity by stunting category. (A-C)** Forest plots of the effects of diarrheal frequency on Shannon’s diversity in the frequency only models (panel **A**), Simpson’s diversity in the frequency only model (**panel B**), and Simpson’s diversity in the full model (panel **C**), Significant differences between groups in post-hoc planned linear contrasts are indicated. *P*-values were corrected by Holm’s method. ^†^*P<0.1;* * *P*<0.05; ** *P*<0.01.

.

**Supplementary tables.**

**Table S1: Amplicon sequence variants (ASVs) and assigned taxonomy.**

**Table S2: Results of linear mixed effects models of bacterial diversity and richness.**

**Sub-table A** shows the results of stepwise model simplification by the Akaike information criterion for each model, presenting the order of elimination of terms and the associated *P*-values. For each metric of diversity and richness, results are given for models including one of diarrheal frequency, duration, severity, or time since last diarrheal episode as predictors. **Sub-tables B-E** present analysis of variance tables for the simplified models for each measure of bacterial diversity and richness, and include the sums of squared errors (SS), mean squared errors (MS), degrees of freedom (DF), *F*-values and *P*-values. **Sub-tables F-I** present coefficient tables for each simplified model. Estimates, standard errors (Std. Errors), degrees of freedom (DF), *t*-ratios, and *P*-values are provided. **Sub-tables J-N** present linear contrasts of means and slopes for breastfeeding categories, stunting categories, and the effects of diarrheal variables and age across stunting categories. **Sub-table O** shows the number of fecal samples in each stunting category in each sampling period.

**Table S3: Results of analyses of associations between specific ASVs and diarrhea, growth, diet, and health variables.**

**Sub-tables A-D** present the results of Poisson models with quasi-GLM correction for the relative abundance of specific ASVs in the gut microbiota of the MAL-ED Peruvian birth cohort at 6, 12, 18, and 24 months of age, respectively. **Sub-tables E-G** present the results of logistic regression models of the presence and absence of ASVs present in the fecal microbiota of children at each sampling time point.

**Table S4: Results of generalized linear models predicting future diarrheal frequency.**

**Sub-tables A, B,** and **C** present coefficients and their standard errors for each negative binomial and Poisson models of diarrheal frequency from 6-12, 12-18, and 18-24 months of age, respectively. The *Z*-values and associated *P*-values are presented for each estimate, as well as *P*-values from likelihood ratio tests between models including and excluding each predictor.
